# 

**Published:** 1892-07-30

**Authors:** 


**Notices of Births, Deaths, and Marriages,** are inserted in this column at a charge of 2s. 6d. for an announcement not exceeding four lines.

### NOTICE TO CORRESPONDENTS.

All MS., Letters, Books for Review, and other matters intended for the Editor should be addressed The Editor, The Lodge, Porchester Square, London, W.

The Editor cannot undertake to return rejected M.S., even when accompanied by stamped directed envelope.

**Notice to Advertisers and Subscribers.**

All Advertisements, Orders for Copies of The Hospital, and Business Communications should be addressed to The Publisher, *not to the Editor*, The Hospital, 140, Strand, W.C.

The Cost of Subscription, post free, is as follows:—Per week, 2½d.; per quarter, 2s. 6d.; per half-year, 5s.; per annum, 8s. 6d. Foreign:—Per annum, 10s. 8d.; payable, in all cases, in advance.

**"Burdett's Hospital Annual," 1891—1892.**

Third year of publication. 600 pp. Boards, gilt lettering. A most complete guide to British and Colonial Hospitals, Dispensaries, Nursing and Convalescent Institutions, and Asylums. Price 5s. 6d. Published by The Scientific Press (Limited), 140, Strand, W.C.

**NOTICE.—The Index for Vol. XI. (ending March, 1892) is on sale at the Office of this Journal, price 2½d., post free.**

### SCRAPS AND GLEANINGS.

THE Warminster Hospital Sunday Fund this year amounted to £40 18s. 8½d.

ABOUT £300 was collected on Hospital Saturday for the Walsall Cottage Hospital.

THE Bury Town Council have under consideration the erection of a new sanitary hospital.

THE Royal Hospital for Incurables, Dublin, has been presented with several pianos for the wards.

AN important meeting was held last week at the Dewsbury Infirmary, when alterations in the medical staff were reported, and the salaries of House Surgeon and Matron increased.

HALIFAX is still labouring under the difficulty of establishing a Combined Districts Infectious Hospital. Each local board seems to be more alarmed lest the others should be the gainers than anxious to secure its own benefits.

THE financial difficulties of the Addenbrooke's Hospital, Cambridge, still continue. A correspondent to the *Cambridge Chronicle* suggests a "people's" collection of a penny a week, for all the surrounding districts, as a means towards substantially increasing the income of the hospital.

AT the annual meeting of the Miller Hospital and Royal Kent Dispensary, the Governors agreed that steps should be taken to build a large hospital, with at least 200 beds, for South-East London, as recommended by the Lords' Committee. A Special Committee was appointed to carry out the work.

A SMALL patient at the Aberdeen Fever Hospital managed to effect his escape through the window during the temporary absence of the nurse from the ward. He was discovered wandering in the rain by a lady, and was returned to the hospital. It is not stated that any serious consequences have been the result of his rash act.

AN interesting gathering assembled at the Princess Alice Hospital, Eastbourne, recently, to inspect the work and progress of the institution. Thirty beds are now available, and several of these have been endowed by generous friends. Some of the pictures which adorn the walls of the wards have been presented by the Queen.

ON Hospital Saturday in Bootle last year an experiment was made of a somewhat original idea. Boxes resembling hospital cots were placed in the streets in conspicuous localities; these realized a sum of £227. This year the experiment has been repeated with even better results, £308 having been received. This happy thought seems worthy of adoption elsewhere.

THE Bristol Medical Missionary Society, which for years has done excellent work amongst the poor, has been opening new premises, which are provided with every convenience. Twenty-five ladies are employed in dispensing and surgical dressing. In the new buildings a class-room has been provided for them, in which they receive instruction from the Medical Superintendent.

MRS. RICKS, the aged negress, who was received by the Queen at Windsor Castle a fortnight ago, was last week brought to the Mansion House by his Excellency the Liberian Minister and Mr. Blyden, and presented to the Lord and Lady Mayoress, with whom she lunched. She afterwards took a short drive in the Lord Mayor's state carriage, much to her evident delight.

DURING the past year 332 in-patients and 3,851 out-patients were treated at the Yarmouth Hospital. At the annual meeting, the thanks of the Committee were tendered to the employés of Messrs. Steward, Pattison, and Co., Messrs. Bly and Sill, and the Locomotive Department of the Railway for their weekly contributions, and they commended the example thus set to other firms.

ACCORDING to the thirtieth report of the Royal Commissioners of the Patriotic Fund, just published, the funds administered by the Commission up to December 31st, 1891, amounted to £764,211, of which £367,935 stands to the credit of the Patriotic Fund, £101,200 to the "Soldiers' Effects" Fund, and £156,852 to the Royal Victoria Patriotic Asylum Endowment Fund. The total number of officers' widows receiving allowances was 95.

AT the opening ceremony of the bazaar recently held at Hampstead in aid of the building fund of the Great Northern Central Hospital, it was announced that a gentleman, who, at his own request, remains for the present anonymous, kindly offered to subscribe £500 to the hospital if nine others would follow his example. The committee have since received two similar promises. Further help is appealed for, as the present number of beds is inadequate to meet the wants of the poor in the district.

THE foundation stone of the new infirmary, Lewisham, was laid by the Bishop of Lichfield. The infirmary will be built with grey stock bricks, with red facings and some Portland stone in front, and will be divided into four blocks, two being allotted to the patients, male and female, the other two being respectively reserved for the administration and staff quarters. All the blocks will be connected by a corridor running down the centre, and they will each be two storeys high except the administration block, which will be only one storey.

THE Duchess of Edinburgh presided yesterday at the annual meeting of the Plymouth and Devonport Branch of the Soldiers' and Sailors' Families' Association, which has for its object the assisting of the necessitous wives and families of all branches of the two services. The Duke of Edinburgh also attended and stated that the thoroughness of the society's organisation was shown in the case of the "Serpent" disaster, when assistance was promptly administered to the bereaved and distressed families of the drowned seamen.

A NEW Isolation Hospital and Disinfector has been erected at Carr Gate by the Wakefield Rural Sanitary Authority. The hospital is of corrugated iron, lined inside with felt and matchwood boarding. There are two wards, each 30 ft. by 20 ft., and the height from the floor to the apex is 17 ft. Each ward contains eight beds. The wards are heated by Shoreland's Manchester stoves, with cold air inlets. In the centre of the building, and between the two wards, is the Matron's room, with a window on either side to overlook both the male and female patients.

### THE STUDENT OF MEDICINE.

[All communications for this Supplement should be addressed to THE EDITOR of THE HOSPITAL, at 140, Strand, with the words, "Students' Column," written in the left hand top corner of the envelope.]

### APPOINTMENTS.

Bannatyne, Gilbert A., M.D.Glasg., appointed Honorary Medical Officer to the Bath Royal United Hospital. Benson, Annette M., M.B., B.Sc.Lond., appointed Resident Medical Officer to the Victoria Hospital for Children, Hull. Evans, A. Ernest, M.B., C.M.Glasg., appointed house physician to the City of London Chest Hospital. Fuller, John R., M.R.C.S., L.R.C.P.Lond., appointed Senior Resident Medical Officer to the Birmingham Children's Hospital. Green, A., M.B.Lond., L.R.C.P., M.R.C.S., appointed Surgeon to the Chesterfield and North-East Derbyshire Hospital. Ninnis, Belgrave, Deputy Surgeon-General, M.D., appointed Principal Medical Officer at Melville Naval Hospital, Chatham. Ormerod, Henry Lawrence, M.R.C.S., L.R.C.P., appointed House Physician to the Bristol Royal Infirmary. Rainy, Harry, M.A., M.B., C.M.Edin., appointed Resident Medical Officer to the Chalmers Hospital, Edinburgh. Richardson, G. Q., L.R.C.P., L.R.C.S.Irel., appointed Junior Resident Medical Officer to the Birmingham Children's Hospital. Saundby, Robert, M.D.Edin., F.R.C.P. Lond., appointed Professor of Medicine in the Queen's Faculty of Medicine, Mason College, Birmingham. Smith, Priestley, M.R.C.S.Eng., appointed Lecturer on Ophthalmology at Mason College, Birmingham. Watson, J., M.B.Durh., F.R.C.S.Edin., L.R.C.P.Lond., appointed House Physician to the Wolverhampton and Staffordshire General Hospital. Willey, Charles Henry, M.D., B.Sc.Edin., M.R.C.S., appointed Honorary Medical Officer to the Children's Hospital, Sheffield. Wilson, Arthur H., M.R.C.S., appointed Honorary Surgeon to the Liverpool Northern Hospital. Vernon, Mr. H., appointed Assistant Medical Superintendent to the Paddington Infirmary.

### VACANCIES.

The following vacancies are announced this week, the amount of salary in each case being given after the name of the institution:

### Resident Medical Officers.

Eccles and Patricroft Hospital, House Surgeon—Salary, £60 per annum, with board, &c.
General Hospital, Birmingham, Two Assistant House Surgeons—board, &c.
Horton Infirmary, Banbury, House Surgeon and Dispenser—Salary, £60 per annum, with board, &c.
Royal Portsmouth, Portsea, and Gosport Hospital, Assistant House-Surgeon, appointment for six months—Board, &c., and honorarium of £15 15s. at the expiration of term of office.

### Non-Resident Medical Officers.

Eccles and District Medical Association, Dispenser.
Liverpool Stanley Hospital, Honorary Physician.
Pharmaceutical Society of Ireland, Examiner in Practical Pharmacy—To hold office for one year, but eligible for re-election annually for four additional years.

### PASS LISTS.

### Royal College of Physicians and Surgeons (*continued*).

Passed in Anatomy and Physiology on Thursday, July 7th: A. Armer, of St. Bartholomew's and Guy's Hospitals; C. F. Gordon, of St. Bartholomew's Hospital; F. J. Hughtrede Cann, of Guy's Hospital; P. G. Mould, of Owen's College, Manchester, and St. Mary's Hospital; G. B. C. Blount, of St. Thomas's Hospital; T. Lambe, of University College; and A. G. L. Smith, of Middlesex Hospital.

Passed in Anatomy only: J. W. Ensor and M. E. Tresidder, of Guy's Hospital; G. W. Brown, and W. Herbert, of St. Thomas's Hospital; A. S. McSorley, of King's College; T. S. Pigg and A. R. H. Skey, of St. Bartholomew's Hospital; F. A. Phillips, of St. George's Hospital; M. D. Blake, of St. George's Hospital and Mr. Cooke's School of Anatomy and Physiology; R. L. Grosvenor, of Cambridge University and St. Mary's Hospital; and W. G. Noble, of London Hospital.

Passed in Physiology only: J. R. Benson, of King's College; J. S. Hosford, E. G. L. Goffe and J. W. Stokes, of University College; E. J. C. Kennedy, of Victoria University, Ontario; W. A. Sharpin, of St. George's Hospital; W. A. Carden, of Guy's Hospital; D. L. Jones and G. G. Oakley, of St. Bartholomew's Hospital; A. Stanley-Matthews, of St. Thomas's Hospital; and A. Carruthers, of St. Thomas's Hospital and Mr. Cooke's School of Anatomy and Physiology.

Nine candidates were referred in both subjects, seven in Anatomy only and nine in Physiology only.

Passed in Anatomy and Physiology on Friday, July 8th: F. Lond, of Guy's Hospital; A. E. Lovitt, W. G. Mortimer, J. H. Power, of London Hospital; R. T. Cæsar, of London Hospital and Mr. Cooke's School of Anatomy and Physiology; M. Wheeler, of Edinburgh University and St. Thomas's Hospital; G. C. Barker, of King's College; W. F. Cross, of St. Batholomew's Hospital; L. H. Callender and R. W. Dodgson, of St. Mary's Hospital; A. H. Waring, of University College.

### The Hospital Advertiser.

### THE STUDENT OF MEDICINE—(*continued*).

Passed in Anatomy only: E. Folliott, W. M. Coghlan, and B. W. Holmes, of St. Bartholomew's Hospital; P. H. Collingwood, J. H. de Villiers, G. Candler, and F. E. Saunders, of St. Thomas's Hospital; N. Macdonald, of St. Mary's Hospital; T. Herbert, of Middlesex Hospital; A. S. Turner, of Guy's Hospital; and A. W. Hayles, of King's College.

Passed in Physiology only: G. H. Goddard and C. F. Wanhill, of University College; and F. E. H. Keogh, of St. Mary's Hospital.

Eleven candidates were referred in both subjects, three in Anatomy only, and eleven in Physiology only.

Passed in Anatomy and Physiology on Saturday, July 9th: C. H. Hunter, H. F. Spon, and E. T. Shorland, of Guy's Hospital; S. H. White, J. B. Brash, and S. Bridger, of University College; F. Copeland, of Westminster Hospital; G. W. Connor and T. H. Wells, of Middlesex Hospital; W. W. Woolliscroft, of Charing Cross Hospital; F. R. S. Cosens and G. A. Leon, of London Hospital.

Passed in Anatomy only: W. P. Brooks and M. M. Lowsley, of Charing Cross Hospital; and A. B. R. Sworn, of University College.

Passed in Physiology only: R. L. Roberts, of Guy's Hospital; T. J. McCulla, of London Hospital and Mr. Cooke's School of Anatomy and Physiology; and J. Blackwood, of University College.

Eighteen candidates were referred in both subjects, three in Anatomy only, and three in Physiology.

Passed in Anatomy and Physiology on Monday, July 11th: C. J. Kearney and W. L. B. Davies, Guy's Hospital; R. D. Fisher, E. C. Smith, and T. P. Littlejohn, London Hospital; J. W. Leake, R. W. S. Christmas, Charing Cross Hospital; S. E. A. Zichy-Wornarski, Melbourne University; M. B. Johnson, Cambridge University and St. Mary's Hospital; J. Hayes, McGill College, Montreal, and Mr. Cooke's School of Anatomy and Physiology; and E. F. Clay, Westminster Hospital.

Passed in Anatomy only: D. N. Maclennan, Queen's University, Kingston, Canada, and Mr. Cooke's School of Anatomy and Physiology; H. E. Dixon and T. W. Turner, London Hospital; L. Savin, Middlesex Hospital; and W. N. Barron, St. Bartholomew's Hospital.

Passed in Physiology only: H. C. Lambart, Queen's College, Birmingham, and Bristol School of Medicine; A. B. Wright and A. S. Grant, London Hospital; J. A. MacPhail, McGill College, Montreal; D. C. Kemp, University College; A. E. Peake and F. Morton, Westminster Hospital; A. R. Hitchfield, Guy's Hospital; A. A. Rogers, Bartholomew's Hospital; and W. Ashford, St. Thomas's Hospital.

Nine candidates were referred in both subjects, five in Anatomy only, and Four in Physiology only.

Passed in Physiology only, on Tuesday, July 12th: H. Harvey, W. P. Thomas, J. J. Spears, and W. F. Adams, London Hospital; M. H. C. Palmer, Cambridge University and London Hospital; J. R. Webb, Melbourne University and London Hospital; R. D. Stacy, W. H. Pope, M. A. Cooke, P. W. G. Shelley, B. M. Hughes, A. N. Wilde, St. Bartholomew's Hospital; W. J. C. Ray and E. Haines, St. Thomas's Hospital; W. Gilbertson, Cambridge University and St. Thomas's Hospital; E. Fisk, W. F. Byford, S. Rivers, T. S. Biggs, and P. E. Tresidder, Guy's Hospital; A. [illegible] Davey and J. Gardiner, Middlesex Hospital; C. Mathews, St. [illegible]ary's Hospital; and D. Fogarty, Ledwich School of Medicine, Dub[illegible] and Mr. Cook's School of Anatomy and Physiology.

Eleven candidates were referred in Physiology only.

### Royal College of Surgeons of England.

The following gentleman, having previously passed the necessary examinations, and having now attained the legal age of twenty-five years, was at the quarterly meeting of the Council on July 14th admitted a Fellow of the College: A. W. W. Lea, M.B.Lond., L.R.C.P.Lond., Owens College and Royal Infirmary, Manchester. Diploma of Member dated August 1st, 1889.

### ATHLETICS.

### United Hospitals A.C. v. Edinburgh University.

Held at Stamford Bridge, July 13th, 1892.

100 Yards.—H. T. Bell, U.H.A.C., first; W. N. Barrow, U.H.A.C., second; G. D. Ross, Edinburgh, third; W. F. Atkinson, Edinburgh, absent.

Three Miles.—H. A. Munro, U.H.A.C., first; G. W. Pollard, Edinburgh, second; S. B. Figgis, Edinburgh, A. N. Wilde and P. Wilmot, U.H.A.C., absent.

440 Yards.—J. W. Summerhayes, U.H.A.C., first; Crosby Hamilton, Edinburgh, second; J. G. Turner, U.H.A.C., third.

High Jump.—R. Williams, Edinburgh, 5 ft. 8 in., first; P. A. Pierre, U.H.A.C., 5 ft. 7 in., second. T. M. Donovan, Edinburgh, and H. A. Pierre, U.H.A.C., also competed.

Putting the Weight.—W. G. West, U.H.A.C., 33 ft. 8 in., first; T. A. Chalmers, Edinburgh, 32 ft. 8½ in., second; C. W. Donald, Edinburgh, 32 ft. 2 in., third. J. S. Mackintosh, U.H.A.C., also competed.

Throwing the Hammer.—T. M. Donovan, Edinburgh, 96 ft. 1 in., first; J. E. Fraser, U.H.A.C., 92 ft. 4 in., second; J. S. Mackintosh, U.H.A.C., 84 ft. 9 in., third. T. A. Chalmers, Edinburgh, also competed.

Half-mile.—P. W. James, U.H.A.C., first; S. B. Figgis, Edinburgh, second; A. Hay, U.A.H.C., 0; W. C. Hamilton, Edinburgh, 0.

220 Yards.—H. T. Bell, U.H.A.C., first; W. G. Richards, U.H.A.C., second; M. Guthrie, Edinburgh, 0; W. F. Atkinson, Edinburgh, 0.

120 Yards Hurdles.—T. M. Donovan, Edinburgh, first; P. R. Lowe, U.H.A.C., second; R. Williams, Edinburgh, third; W. Kent-Hughes, U.H.A.C., 0.

One Mile.—H. A. Monro, U.H.A.C., first; G. W. Pollard, Edinburgh, second; M. E. Tressider, U.H.A.C., third; W. Kent-Hughes, U.H.A.C., 0; S. B. Figgis, Edinburgh, 0.

Long Jump.—A. P. Square, U.H.A.C., 21 ft. 10½ in.; T. M. Donovan, Edinburgh, 19 ft. 2½ in.; J. S. Mackintosh, U.H.A.C., 18 ft. 11¾ in. M. Guthrie, Edinburgh, also competed.

